# Is there any difference between endometrial hyperplasia and endometrial carcinoma in terms of expression of TRPM2 and TRPM7 ion channels?

**DOI:** 10.3906/sag-1810-176

**Published:** 2019-04-18

**Authors:** Emre YALÇIN, Şehmus PALA, Remzi ATILGAN*, Tuncay KULOĞLU, Ebru ÖNALAN, Gökhan ARTAŞ, İlay BURAN

**Affiliations:** 1 Department of Obstetrics and Gynecology, School of Medicine, Fırat University, Elazığ Turkey; 2 Department of Histology and Embryology, School of Medicine, Fırat University, Elazığ Turkey; 3 Department of Department of Medical Biology, School of Medicine, Fırat University, Elazığ Turkey; 4 Department of Pathology, School of Medicine, Fırat University, Elazığ Turkey

**Keywords:** TRPM2, TRPM7, endometrial hyperplasia, endometrioid adenocarcinoma

## Abstract

**Background/aim:**

This study compared TRPM2 and TRPM7 ion channel gene expression and immunohistochemical staining in endometrial hyperplasia and endometrium adenocarcinoma.

**Materials and methods:**

Sections were taken from paraffin blocks of 120 patients who were divided into 6 groups as follows: G1 (n = 20), proliferative endometrium (PE); G2 (n = 20), EH without atypia; G3 (n = 20), EH with atypia; G4 (n = 20), stage 1A, grade 1 EC; G5 (n = 20), stage 1A, grade 2 EC; and G6 (n = 20), stage 1A, grade 3 EC. TRPM2 and TRPM7 genes were analyzed with qRT-PCR in paraffin-embedded tissue samples. Under light microscopy, TRPM2 and TRPM7 immunostaining scores of the samples taken from polylysine slides were evaluated.

**Results:**

Compared to G1, TRPM2 mRNA gene expression was significantly downregulated in G3 and G5. TRPM2 immunoreactivity scores were similar in all groups. TRPM7 mRNA gene expression was significantly downregulated in G2, G3, and G6 when compared to G1. TRPM7 immunoreactivity scores were similar in G1, G2, and G3, but significantly decreased in G4, G5, and G6.

**Conclusion:**

Reduction in TRPM7 ion channel activity may be a progression marker for endometrial hyperplasia regardless of the atypical criteria.

## 1. Introduction

Endometrial hyperplasia (EH) is a pathologic condition characterized by hyperplastic changes in the endometrial glandular and stromal structures covering the uterine cavity (1). Chronic exposure to estrogens not met by progesterone is considered as a key component in the development of EH and endometrial carcinoma (EC) (2). The important risk factors for EH development are obesity, unbalanced estrogen therapy, tamoxifen therapy, polycystic ovary syndrome, and nulliparity (3). EH is the precursor for endometrioid adenocarcinoma of the endometrium. According to classical classification, the progression of carcinoma is 0%–3% in simple hyperplasia without atypia, 0%–8% in simple atypical hyperplasia, and 9%–29% in complex atypical hyperplasia (4,5). 

EC is the most common gynecological cancer observed in developed countries (6). EC has two major types: endometrioid carcinoma (type 1) due to hormonal imbalance and serous carcinoma (type 2) not associated with estrogen (7). While EC constitutes 3.6% of all new cancer cases in 2016, it is responsible for 1.8% of all cancer deaths in the United States (8). Moreover, as risk factors including obesity and progressive age become more common, the incidence is likely to increase. Fortunately, based on signs and symptoms (such as abnormal uterine bleeding), an early stage (stage I) diagnosis can be made in many patients (about 75%) (9).

Tumor progression occurs as a result of changes in physiological processes such as cell proliferation, apoptosis, migration, invasion, and angiogenesis. These processes are under the control of the calcium homeostasis and transient receptor potential (TRP) cation channels (10). Ion channels and significant TRP channels are involved in many physiological processes. These channels have been shown to be associated with some serious diseases, such as cancer. During the onset and progression of cancer, altered expression of one or more TRP proteins is of importance (11). Excessive TRPM2 levels in cancer cells may have an enzymatic function in relation to cell proliferation (12). 

Vascular endothelial growth factor (VEGF), basal fibroblast growth factor (bFGF), and platelet-derived growth factor (PDGF) stimulate angiogenesis by increasing the proliferation and migration of endothelial cells. These angiogenic factors may increase intracellular Ca2+ concentration in endothelial cells through the activation of plasma membrane receptors and the release of Ca2+ from the endoplasmic reticulum. This also leads to activation of Ca2+ channels in the plasma membrane. Several studies have emphasized the importance of TRP channel-mediated Ca2+ signals in the angiogenesis process (13).

In previous studies, TRPM2 has been shown to alter endothelial cell function in response to oxidative stress. Blocking TRPM7 has been shown to cause increased growth and proliferation in addition to causing increased expression of nitric oxide synthase and nitric oxide production (14). 

TRPM7 plays a role in angiogenesis and oxidative stress-related cell death (15). Recent TRPM7 studies on ovarian cancer have shown that TRPM7 is necessary for cancer cell growth, migration, and invasion (16).

The purpose of the present study was to investigate the activity of TRPM2 and TRPM7 ion channels due to their close relationship with proliferation and migration mechanisms in endometrial hyperplasia and early stage endometrial cancer.

## 2. Materials and methods

### 2.1. Patient selection

In this retrospective study, qRT-PCR analysis and immunohistochemical staining were performed for paraffin-embedded archive tissues of patients with proliferative endometrium (PE), EH, and early stage EC. Approval to conduct the study was obtained from the local ethics committee of Fırat University (Dated 20.04.2017, Session No: 07, Decision No: 01). Patients diagnosed with PE, EH, and endometrial adenocarcinoma (EC) with hysterectomy between 2007 and 2017 in the Fırat University Faculty of Medicine’s Obstetrics and Gynecology Department were included in the study. The paraffin blocks of 120 patients were resectioned in the pathology clinic. In this study, because it was aimed to investigate the importance of ion channels in the progression from PE to EH and EC, early stage EC cases were selected in the endometrial carcinoma group. A total of 120 cases were divided into 6 groups: G1 (n = 20), PE group; G2 (n = 20), EH without atypia; G3 (n = 20), EH with atypia; G4 (n = 20), stage 1A according to FIGO staging (tumor size <2 cm, myometrial invasion <1/2), grade 1 EC; G5 (n = 20), stage 1A, grade 2 EC; G6 (n = 20), stage 1A, grade 3 EC.

### 2.2. RNA isolation, complementary DNA (cDNA) synthesis, and quantitative real-time PCR (qRT-PCR) analysis 

For RNA isolation from the supplied paraffin blocks, sections of 0.2 mm in thickness were taken, stacked in Eppendorf tubes, and stored at –80 °C for isolation. The RNA isolation from the paraffin blocks was performed using TRIzol. For paraffin melting, sections of 20 µm were placed in Eppendorf tubes and kept for 1 h in a heat block at 65 °C. For the removal of the paraffin, 1000 µL of xylol (heated at 65 °C in an incubator) was placed in Eppendorf tubes, and after 5 min of incubation at 65 °C, centrifugation was performed at 14,000 rpm for 2 min. After the centrifugation, the supernatant was removed. The processes after the xylol addition were repeated 3 times. By placing 1000 µL of 100% ethanol into the Eppendorf tubes, vortexing was done. After holding this mixture for 15 min in the incubator at 37 °C, centrifugation was performed at 14,000 rpm for 2 min and the supernatant was removed. By placing 1000 µL of 70% ethanol into the Eppendorf tubes, vortexing was done. It was held for 15 min at 37 °C in the incubator. Centrifugation was performed at 14,000 rpm for 2 min and the supernatant was removed. By placing 1000 µL of 50% ethanol into the Eppendorf tubes, vortexing was done. It was held for 15 min at 37 °C in the incubator and then centrifugation was performed at 14,000 rpm for 2 min and the supernatant was removed. After that, 750 µL of TRIzol, 60 µL of proteinase K, and 2 µL of RNase inhibitor were added to the Eppendorf tubes and they were held for 1 night in a shaking water bath set to 60 °C. Samples were expected to fall to room temperature. Chloroform (300 µL) was then added to the Eppendorf tubes and vortexing was performed for 15 s. Centrifugation was performed at 4 °C and 20,000 rpm for 20 min and the phase remaining on the top was transferred to new tubes. Isopropyl alcohol (800 µL) was then added to them, and after shaking, the tubes were held for 1 night at room temperature. Centrifugation was performed at 4 °C and 13,500 rpm for 10 min and the upper phase was removed. Washing was done with 75% ethyl alcohol. After shaking, centrifugation was performed at 4 °C and 7500 rpm for 5 min and then tubes were held open for 10 min to allow the release of ethanol. Based on the size of the white RNA pellet remaining at the bottom, DNase, RNase, and pyrogen-free water were added.

Total RNA isolation from tissue samples was performed using TRI Reagent (BioShop, Canada) and the RNA quantity and quality were determined by BioSpec-nano (Shimadzu, Japan). Complementary DNA synthesis was carried out in accordance with the protocol of a High-Capacity cDNA Reverse Transcription Kit (Applied Biosystems, USA) in a thermal cycler (Veriti 96-Well Thermal Cycler, Applied Biosystems). With the obtained complementary DNAs, gene expression levels were determined using an Applied Biosystems 7500 Real Time PCR instrument for transient receptor potential cation channel subfamily M member 2 (TRPM2) and TRPM7 (rabbit anti-TRPM2 antibody, Ab101738, and goat anti-TRPM7 antibody, ab 729, Abcam, Cambridge, UK, Catalog No. QT01609965, QIAGEN). By using EvaGreen 2X qPCR MasterMix (abm), the mixture required for the determination of gene expressions was prepared according to the relevant protocol. Glyceraldehyde 3-phosphate dehydrogenase (GAPDH) (Cat. No. QT00079247, QIAGEN, USA) was used as the control gene (housekeeping). Calculation of differences between gene expressions was performed by the 2–ΔΔCT method.

### 2.3. Immunohistochemical evaluation 

The sections taken from paraffin blocks of 4–6 µm in thickness were taken into polylysine slides. For antigen retrieval, the deparaffinized tissues were passed through a graded alcohol series and boiled in a microwave (750 W) for 7 + 5 min at pH 6 in citrate buffer solution. After boiling, the tissues that were left to cool at room temperature for about 20 min were incubated for 5 min with hydrogen peroxide block solution in order to prevent endogenous peroxidase activity after washing for 3 × 5 min with phosphate buffered saline (PBS) solution [PBS (10 mM Na2HPO4, 10 mM KH2PO4, 0.9 g NaCl/100 mL, pH 7.4), P4417, Sigma-Aldrich, USA), TA-125-HP, Lab Vision Corporation, USA]. 

After washing with PBS for 3 × 5 min, to be able to block the background stain, a solution of Ultra V Block (TA-125-UB, Lab Vision Corporation) was applied to the tissues for 5 min and then they were incubated with 1/200 diluted primary antibodies (rabbit anti-TRPM2 antibody, Ab101738, and goat anti-TRPM7 antibody, ab 729, Abcam) for 60 min at room temperature. The tissues were washed with PBS for 3 × 5 min after the application of the primary antibody and then washed twice with secondary antibodies (biotinylated goat anti-polyvalent, TP-125-BN, Lab Vision Corporation, and donkey anti-goat, sc-2042, Santa Cruz Biotechnology, USA) for 30 min at room temperature in a humidified environment. The tissues were washed with PBS for 3 × 5 min after the application of the secondary antibody and incubated in a humidified environment for 30 min with streptavidin-peroxidase (TS-125-HR, Lab Vision Corporation), and then they were put into PBS. After a solution of 3-amino-9-ethylcarbazole (AEC) substrate + AEC chromogen (AEC substrate, TA-015 and HAS, AEC chromogen, TA-002-HAC, Lab Vision Corporation) was added to the tissues and an image signal was obtained with a light microscope, the tissues were simultaneously washed with PBS. Rabbit IgG was used for the negative control. Tissues counterstained with Mayer’s hematoxylin were treated with PBS and distilled water and covered with appropriate closure solution (Large Volume Vision Mount, TA-125-UG, Lab Vision Corporation). The preparations were evaluated and photographed with a Leica DM500 microscope (Leica DFC295). Under light microscopy, based on the extent of immunoreactivity (0.1: <25%, 0.4: 26%–50%, 0.6: 51%–75%, 0.9: 76%–100%) and the intensity (0: no, + 0.5: very little, + 1: less, + 2: moderate, + 3: severe), the histoscore was determined (histoscore = extensity × intensity) (17).

### 2.4. Statistical analysis 

All analyses were performed with SPSS 22.0 (IBM Corp., Armonk, NY, USA). The data obtained from qRT-PCR evaluations were determined as mean ± standard deviation. 

Immunohistochemistry scores were calculated as median and minimum–maximum values. Whether or not the numerical measurements ensured the normality (normal distribution) assumption was tested by the Kolmogorov–Smirnov test. For numerical measurements that did not show normal distribution, the Mann–Whitney U test was used for the comparison between the two groups. The Kruskal–Wallis test was used for the general comparison between more than two groups. In such cases, for the values that were found significant, Bonferroni correction was performed for the binary comparisons of the groups. P < 0.008 was accepted as statistically significant.

## 3. Results

### 3.1. Genetic findings 

**Figure 1:**
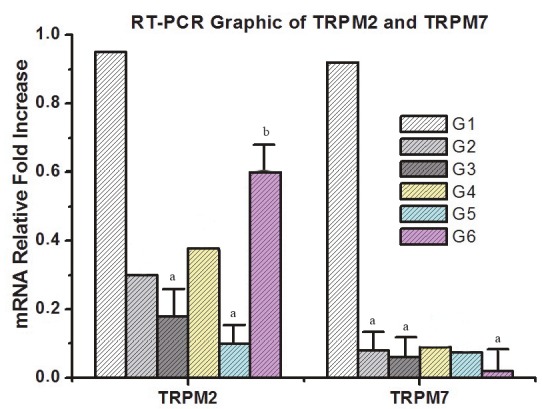
RT-PCR results for TRPM2 and TRPM7. a: Significant downregulation of TRPM2 gene expression is shown in G3 and G5 when compared with G1. b: Statistically significant upregulation of TRPM2 gene expression was observed in G6 when compared to G2. c: Significant downregulation of TRPM7 gene expression is shown in G2, G3, and G6 when compared with G1. G1 = Proliferative endometrium; G2 = endometrial hyperplasia without atypia; G3 = endometrial hyperplasia with atypia; G4 = stage 1A, grade 1 endometrium adenocarcinoma; G5 = stage 1A, grade 2 endometrium adenocarcinoma; G6 = stage 1A, grade 3 endometrium adenocarcinoma.

In terms of gene expression of TRPM2 mRNA between the groups, there was no significant difference between G2 (P = 0.037), G4 (P = 0.047), and G6 (P = 1.00) compared to G1. When compared to G1, a significant downregulation was observed between G3 (P = 0.003) and G5 (P = 0.001). In addition, in within-group comparisons, a statistically significant upregulation was observed in G6 (P = 0.008) when compared to G2. TRPM2 gene expression did not show any significant difference in other within-group comparisons (Figure 1).

In terms of gene expression of TRPM7 mRNA between the groups, a statistically significant downregulation was observed in G2 (P = 0.003), G3 (P = 0.002), and G6 (P = 0.000) compared to G1. There was no significant difference between G1 and G4 (P = 0.01) and G5 (P = 0.012). In addition, in terms of TRPM7 gene expression, no significant difference was observed in the other within-group comparisons (Figure 1).

### 3.2. Immunohistochemical findings

**Figure 2:**
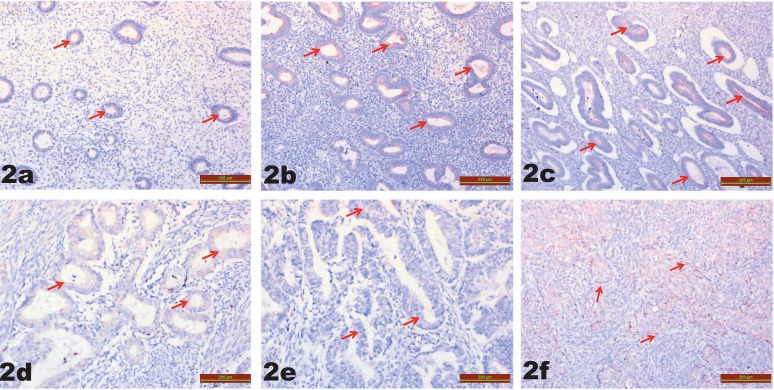
TRPM2 immunostaining is similar in all groups (red arrow): a) G1, b) G2, c) G3, d) G4, e) G5, f) G6. G1 = Proliferative endometrium; G2 = endometrial hyperplasia without atypia; G3 = endometrial hyperplasia with atypia; G4 = stage 1A, grade 1 endometrium adenocarcinoma; G5 = stage 1A, grade 2 endometrium adenocarcinoma; G6 = stage 1A, grade 3 endometrium adenocarcinoma.

Related to TRPM2 immunoreactivity, in the between-group comparison, there was no significant difference for G1 [0.23 (0.1–0.8)], G2 [0.30 (0.1–0)], G3 [0.32 (0.1–0.9)], G4 [0.35 (0.1–1.2)], G5 [0.31 (0.1–0.6)], and G6 [0.34 (0.1–0.8)] (P = 0.1997) (Table; Figure 2). 

**Table:** TRPM2 and TRPM7 immunoreactivity histoscores (extensity × intensity) in all groups.

Groups	TRPM2	TRPM7
G1	0.23 (0.1–0.8)	0.66 (0.1–2.7)
G2	0.30 (0.1–0.6)	0.61 (0.1–1.2)
G3	0.32 (0.1–0.9)	0.65 (0.1–1.8)
G4	0.35 (0.1–1.2)	0.18 (0.0–0.5)a
G5	0.31 (0.1–0.6)	0.19 (0.0–0.5)a
G6	0.34 (0.1–0.8)	0.17 (0.0–0.4)a
P-value	<0.197	<0.001

a: When compared with G1, the TRPM7 immunoreactivity had significantly decreased in G4, G5, and G6 (P < 0.001). G1 = Proliferative endometrium; G2 = endometrial hyperplasia without atypia; G3 = endometrial hyperplasia with atypia; G4 = stage 1A, grade 1 endometrium adenocarcinoma; G5 = stage 1A, grade 2 endometrium adenocarcinoma; G6 = stage 1A, grade 3 endometrium adenocarcinoma.

**Figure 3:**
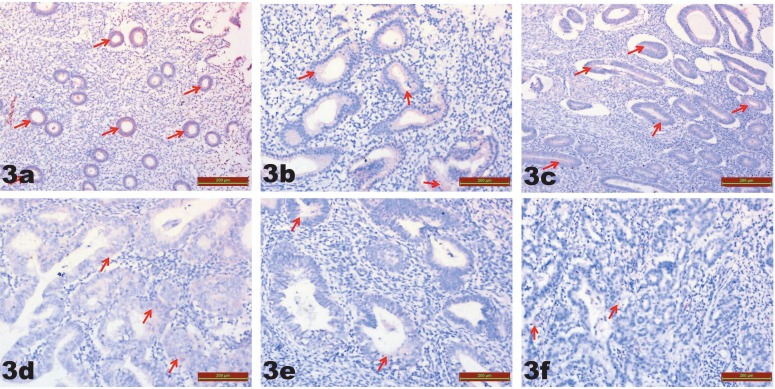
TRPM7 immunostaining is shown as similar in G1, G2, and G3. Decreased TRPM7 immunostaining is shown in G4, G5, and G6 relative to G1 (red arrows). a) G1, b) G2, c) G3, d) G4, e) G5, f) G6. G1 = Proliferative endometrium; G2 = endometrial hyperplasia without atypia; G3 = endometrial hyperplasia with atypia; G4 = stage 1A, grade 1 endometrium adenocarcinoma; G5 = stage 1A, grade 2 endometrium adenocarcinoma; G6 = stage 1A, grade 3 endometrium adenocarcinoma.

Related to TRPM7 immunoreactivity, in the between-group comparison, there was no significant difference for the comparison of G1 [0.66 (0.1–2.7)], G2 [0.61 (0.1–1.2)], and G3 [0.65 (0.1–1.8)] (P > 0.008). When compared with G1 [0.66 (0.1–2.7)], the TRPM7 immunoreactivity had significantly decreased in G4 [0.18 (0.0–0.5)], G5 [0.19 (0.0–0.5)], and G6 [0.17 (0.0–0.4)] (P < 0.001) (Table; Figure 3).

## 4. Discussion

In this study, mRNA gene expression and immunohistochemical staining scores of TRPM2 and TRPM7 were assessed in the course of EH up to the early stage of EC. The results of the study suggest that a significant reduction in TRPM7 mRNA downregulation and immunoreactivity score, especially in the prognosis of EH, may be a progression marker. 

Biomarkers are defined as properties “that can be measured and evaluated objectively as indicators of normal biological and pathogenic processes or pharmacological responses to therapeutic intervention” (18). The best molecular biomarker is a biomarker that is reliable and reproducible and can allow the differentiation of normal/benign, premalignant, and malignant endometrium to ensure that the transition between these three groups is demonstrated or predicted. Until now, there has not been a single candidate that fulfills this role and studies are ongoing. A variety of immunohistochemical biomarkers have been investigated in the literature to help in the diagnosis and classification of EH and to predict the likelihood of transition from EH to EC (19). For example, in their study, Horrée et al. (20) showed that progesterone receptor-B (PR-B) expression was higher in women with atypical hyperplasia with 90% reduced lesion persistence/progression risk. Erkanli et al. (21) showed statistically significant COX-2 overexpression in EH and EC cases compared with PE. Horrée et al. (22) determined that almost all negative cells in inactive endometrium showed increasing expression of p53 in EH where only a few cells were positive and that the highest gene expression was seen in endothelial cells. Kurdoğlu et al. (23) showed a gradual increase in lamin receptor-1 expression in epithelial basal membranes of hyperplastic endometrium, with atypia or not, based on the progression of the disease. They also showed that it may play an important role in the transition from premalignant status to endometrial lesions in malignant conditions. 

The TRP superfamily is a representative of an alliance composed of mostly nonselective Ca2+-permeable cation channels that respond to a wide range of chemical and physical stimuli (24) and represent the potential key players in intercellular signaling between uterine epithelium and stromal cells. However, available information related to the distribution of TRP channels in human endometrial epithelial and stromal cells is insufficient (25). Interestingly, the characteristic mRNA expression profile of some TRP channels throughout the menstrual cycle may suggest that these TRP channels are regulated by sex hormones such as estrogen and progesterone. Regulation of TRP mRNA by steroid hormones in endometrial cells has been previously reported for TRPV6 and TRPC1 (26,27). TRP channels are expressed differentially in different cancer types. It has been reported that TRPM2 is expressed in many cancer types such as bladder, breast, lung, liver, head, and neck cancers (28). Although the mechanisms regulating its expression are not well known, TRPM2 has been shown to have many methylation sites capable of modulating malignancy (29). In our study, it was observed that TRPM2 miRNA gene expression was significantly downregulated in atypia EH and grade 2 EC compared to PE, and that there was a statistically significant upregulation in grade 3 EC compared to EH without atypia; however, there was no significant difference in immunoreactivity staining scores between the groups. The reason why the TRPM2 miRNA downregulation cannot be demonstrated immunohistochemically may be that the gene expression is not at a level that can produce immunohistochemical differences in the amount of suppressed protein. The discrepancy between PCR results and immunohistochemistry results can be explained as follows: in general, sensitivity and specificity increase with PCR methods compared to immunohistochemistry. However, the correlation between these two methods is weak. Each of them performs different analyses in terms of the characteristics of cell biology. The purpose of RT-PCR is to confirm whether the protein-producing gene is active or not through mRNA analysis. On the other hand, immunohistochemistry confirms the presence of protein. The morphology is maintained in the immunohistochemistry procedure and allows recognition of immunostaining heterogeneity and confirmation that the defined positivity is present in tumor cells. On the other hand, the RT-PCR technique is a nonmorphological technique and the contamination of tumor mRNA with normal tissue can affect the results and may cause inconsistencies between the two techniques. As a result, the expression at mRNA and protein levels may not always be synchronized (30,31). In their study, Hiroi et al. (29) showed that the TRPM2 gene was an estrogen-dependent gene in cultured endometrial stromal cells. They also showed that there was no increase in TRPM2 mRNA expression in the medium and late endometrial proliferative phase where estrogen was the highest. They considered that other regulatory factors could be effective on the proliferative endometrial phase of in vivo mRNA transcription in the human endometrium and found that TRPM2 mRNA expression significantly increased in the late secretory phase. In cultured endometrial stromal cells, estrogen and progesterone treatment increased the TRPM2 mRNA expression. In a study conducted on rats, Ahn et al. (32) showed that TRPM2 mRNA levels increased significantly in proestrus and returned to basal levels in metestrus and to previous levels in diestrus. Another reason for the downregulation of the TRPM2 mRNA expressions in our grade 2 EC and EH with atypia groups and the upregulation in grade 3 EC compared to EH without atypia may be the fact that, as mentioned above, estrogen is different in its effects on the endometrium at different periods and many factors coexist in vivo in the etiopathogenesis of carcinoma. More extensive research is needed in this regard. Increased evidence has shown that TRPM7 members play an important role in cellular processes, embryonic development, and human disease, especially in cancer. Accumulated data emphasize the potential importance of TRPM7 as a molecular biomarker and therapeutic target in human malignancies (33–36). TRPM7 is both an ion channel and a kinase protein that regulates different cellular processes in many solid tumor types. However, EC has not been evaluated yet. Nakashima et al. (37) demonstrated that immunohistochemical and siRNA suppression of TRPM7 promotes proliferation, migration, and invasion in esophageal squamous cell carcinoma. Conversely, Rybarczk et al. (38) reported that high TRPM7 levels were associated with poor prognosis in ductal adenocarcinoma of the pancreas. Wang et al. (16) suggested that overexpression of TRPM7 in ovarian cancer is associated with poor prognosis. In this study, we also showed that TRPM7 miRNA was downregulated in the EH (with atypia or without atypia) and grade 3 EC groups compared to the PE group. Immunohistochemically, we found only a significant decrease in the EC groups (grades 1, 2, and 3). However, we could not find any significant difference between grades 1, 2, and 3 in stage 1A EC. When we compared our genetic results and immunohistochemical results, we observed that there was a similarity only in the grade 3 EC group.

TRPM7 has been reported to play a critical role in the transition from stagnation to proliferation (39). Throughout the menstrual cycle, stromal cells go to proliferation and differentiation. It has been shown that TRPM7 plays a critical role in proliferating cells and gene expression decreases in differentiated cells (40,41). Sahni and Scharenberg (42) reported that the growth rates of TRPM7-deficient cells are rapidly downregulated, which leads to a secondary arrest in proliferation. On the other hand, the effect of TRPM7 on cell survival and growth has been investigated and silencing of TRPM7 has been shown to cause growth and proliferation for some cell types such as human umbilical cord endothelial cells (14). Although TRPM7 mRNA gene expressions were significantly downregulated in EH (with or without atypia), the TRPM7 immunoreactivity score in our study was not different from the PE group even in the atypical EH group. Thus, we can say that the different mechanisms that play a role in cancer biology may contribute to the reduction of TRPM7 immunoreactivity in our EC groups. Nakashima et al. (37) also showed that TRPM7 downregulation significantly increased cell proliferation, migration, and invasion. This suggests that the significant reduction in TRPM7 immunoreactivity in our EC groups may be a consequence of the proliferation and the increase in invasion compared to EH groups. The fact that the TRPM7 gene expression in grade 3 EC is significantly downregulated compared to grade 1 and grade 2 can also provide insight for future studies by indicating that TRPM7 may be a sign of progress in EC. 

The main limitation of our study is that it is a retrospective study with a limited number of cases. It may also have been more valuable to examine cases progressing to stage 1A EC by monitoring EH cases in the long term. However, it is clear that this would not be ethical. On the other hand, the fact that TRPM2 and TRPM7 have been studied for the first time in EH and EC as progression markers can be shown as the strength of our study.

In conclusion, in early stage endometrial cancers, a significant reduction in TRPM7 ion channel activity relative to endometrial hyperplasia may be a progression marker for endometrial hyperplasia independently of the atypia criteria.

## Acknowledgment

This study was funded by Scientific Research Grants Unit of Fırat University (grant number: TF.17.13).
